# Performance enhancement of ITO/oxide/semiconductor MOS-structure silicon solar cells with voltage biasing

**DOI:** 10.1186/1556-276X-9-658

**Published:** 2014-12-05

**Authors:** Wen-Jeng Ho, Min-Chun Huang, Yi-Yu Lee, Zhong-Fu Hou, Changn-Jyun Liao

**Affiliations:** 1 Department of Electro-Optical Engineering, National Taipei University of Technology, No. 1, Sec. 3, Zhongxiao E. Road, Taipei 10608, Taiwan

**Keywords:** Conversion efficiency, ITO, Metal oxide semiconductor, Silicon solar cell, Voltage biasing

## Abstract

In this study, we demonstrate the photovoltaic performance enhancement of a p-n junction silicon solar cell using a transparent-antireflective ITO/oxide film deposited on the spacing of the front-side finger electrodes and with a DC voltage applied on the ITO-electrode. The depletion width of the p-n junction under the ITO-electrode was induced and extended while the absorbed volume and built-in electric field were also increased when the biasing voltage was increased. The photocurrent and conversion efficiency were increased because more photo-carriers are generated in a larger absorbed volume and because the carriers transported and collected more effectively due to higher biasing voltage effects. Compared to a reference solar cell (which was biased at 0 V), a conversion efficiency enhancement of 26.57% (from 12.42% to 15.72%) and short-circuit current density enhancement of 42.43% (from 29.51 to 42.03 mA/cm^2^) were obtained as the proposed MOS-structure solar cell biased at 2.5 V. In addition, the capacitance-volt (C-V) measurement was also used to examine the mechanism of photovoltaic performance enhancement due to the depletion width being enlarged by applying a DC voltage on an ITO-electrode.

## Background

To face the threat of global warming caused by fossil fuel-based energy consumption, researchers have been encouraged to search for a viable form of energy that generates minimal CO_2_ emissions. Photovoltaic energy can provide a good option as a renewable source in the future because it is a clean form of energy. However, the cost per unit of electricity generated from a photovoltaic system is higher than the retail price of electricity generated by more conventional means today. Presently, the dominant photovoltaic technology is based on bulk wafer-based crystalline silicon (Si) technology. Thus, reducing the cost of Si material would be one of the first options to consider in attempting to reduce the cost of electricity generated by photovoltaic systems. However, while the cost of bulk materials used in these photovoltaic cells has steadily decreased over the past 10 years, this trend cannot continue indefinitely. On the other hand, researchers have also been trying for decades to improve the efficiency of photovoltaic devices and reduce the cost of their fabrication by using novel device structures involving relatively simple fabrication methods. In fact, a number of alternate structures have been used to achieve higher efficiency, including hetero-junction [1-3], multi-junction [4-6], metal-insulator-semiconductor (MIS) [7-9], and metal-oxide-semiconductor (MOS) solar cells [10-12]. MIS silicon solar cells are a promising candidate for the cost-effective photovoltaic devices due to the low temperature device process. The best MIS inversion-layer (MIS-IL) silicon solar cells are fabricated on p-Si substrate using an Al/SiOx/p-Si MIS tunnel contact and a SiNx/Cs ions/SiOx surface passivation and antireflection coating between the front finger electrodes [13]. The photocurrents generated in MIS solar cells tunnel through a thin oxide layer to the Al electrode, and a high-quality thin oxide layer is required. Moreover, compared with diffused p-n junction solar cells, issues related to the critical Cs-treated processing and long-term instability interface states in MIS solar cells still need to be resolved [8,9]. Furthermore, while the simulation and optimization of MIS-IL Si solar cells has been previously reported on [14], only a few studies of the use of voltage biasing effects on the MIS- or MOS-solar cells to enhance the photovoltaic efficiency have been conducted [15-18].

In this study, the novel MOS-structure silicon solar cell consisted of a conventional p-n junction semiconductor, and a transparent-antireflective ITO/oxide film was fabricated on the p-n semiconductor. The voltage biasing effects on the enhancement of photovoltaic performance were investigated, and photovoltaic performance enhancements achieved by application of the biasing voltage on the ITO-electrode were confirmed and examined using photovoltaic current-voltage (I-V) and capacitance-voltage (C-V) measurements.

## Methods

The 400-μm-thick, (100) oriented, 1- to 10-Ω-cm, p-type (boron-doped), and double-sided polished Si wafer was first cut into small samples with the dimensions of 1 × 1 cm^2^ for bare silicon solar cell fabrication. After RCA cleaning, all of the Si samples were coated with phosphorus liquid source (provide by Emulsitone Chemicals LLC., Washington, NJ, USA) using a spin-on film (SOF) technique. In the SOF processing, all samples were spun at a speed of 6,000 rpm for 20 s, followed by a prebaking process on a hot plate at 200°C for 5 min for solvent removal and 400°C for 10 min for cross-linking. Next, the samples were capped with a 200-nm-thick SiO_2_ layer using e-beam evaporation and heated in a rapid thermal annealing (RTA) chamber in an N_2_ atmosphere at 900°C for 2 min to implement the phosphorus diffusion to obtain an n^+^-Si emitter. After diffusion, the samples were soaked in an HF solution to remove capped SiO_2_ and the phosphorus oxide layer. The phosphorus diffusion profile was confirmed by a secondary ion mass spectrometry (SIMS) measurement on a single test sample. Next, the samples were isolation-etched using KOH solution through a photolithograph process to obtain 4 × 4 mm^2^ individual areas. Finally, a 200-nm-thick Al film and a 20-nm-Ti/200-nm-Al film were respectively deposited on the backs and fronts of the samples using e-beam evaporation, forming front and back electrodes. After being annealed in an RTA chamber at 450°C for 15 min in an N_2_ atmosphere, a bare solar cell (the reference cell-1) with ohmic contact electrodes was obtained.

**Figure 1:**
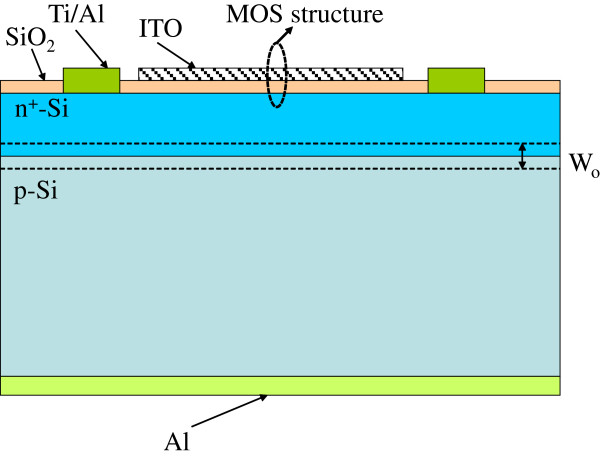
Schematic of an ITO/oxide/p-n-semiconductor (MOS-structure) silicon solar cell.

Before fabricating MOS-structure solar cells, it must be confirmed that the optical and electrical properties of SiO_2_, ITO, and SiO_2_/ITO films are suitable for the applications of MOS-structure solar cells. Therefore, the breakdown field of the e-beam deposited SiO_2_ film, the conductivity and transparence of the thermal sputtered ITO film, and the reflectivity of SiO_2_/ITO film must be measured and characterized. In this study, ITO film deposited on a glass substrate using a thermal sputtering deposition at 250°C in Ar ambient exhibited conductivity of >4.637 × 10^7^ μs/cm, transmittance of >80% at the visible wavelength range, and a refractive index of about 1.87. The breakdown field of the deposited SiO_2_ film was >5 × 10^6^ V/cm (*I*_0_ < 10 μA at 25°C). Finally, a 10-nm-thick SiO_2_ film and a 50-nm-thick ITO film were deposited subsequently by e-beam evaporator and thermally sputter on the surface of a bare diffused p-n junction silicon solar cell), and the MOS-structure solar cell was thus obtained (the reference cell, zero biasing). Figure 1 shows the schematic of an ITO/oxide/p-n-semiconductor (MOS-structure) silicon solar cell. The active area of a MOS-structure solar cell was approximately 0.1437 cm^2^.

To characterize the performance of the MOS-structure solar cell, the optical reflectance, external quantum efficiency (EQE), photovoltaic current-voltage (I-V) under one-sun AM 1.5G illumination (100 mW/cm^2^, 25°C) and capacitance-voltage (C-V) of the p-n junction of the MOS-structure solar cell without application of a biasing voltage on the ITO electrode (at 0 V) were measured as a reference capacitance. Furthermore, the photovoltaic I-V and C-V values of the MOS-structure solar cell with applied biasing voltages from 0 to -2.5 V on the ITO electrode were measured and compared to the reference data. The p-n junction capacitance decreased and the short circuit current (*I*_sc_) increased as the biasing voltage on the ITO electrode was increased, indicating that the depletion width under the ITO region was enlarged and that the photo-carriers generated, transported, and collected photo-carriers more effectively due to the biasing effects.

## Results and discussion

**Figure 2:**
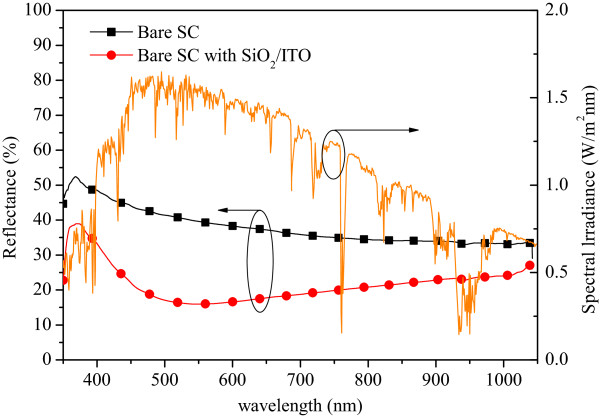
Reflective spectrums of the bare solar cell and the MOS-structure solar cell.

**Figure 3:**
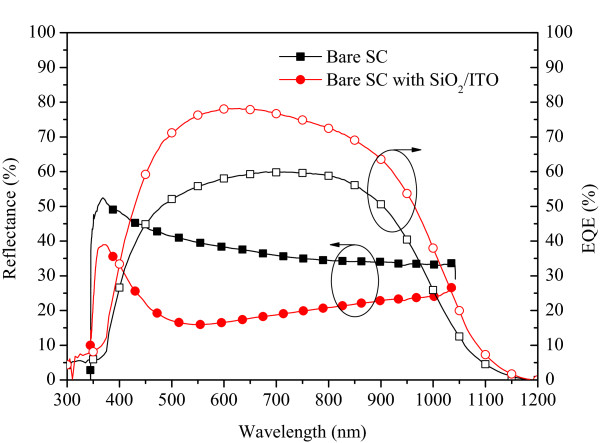
**EQE responses of the bare solar cell and the MOS-structure solar cell with a 50-nm ITO/10-nm SiO**_**2 **_**AR coating.**

The reflective spectrums of the bare solar cell and the MOS-structure solar cell are shown in Figure 2. The AM 1.5G solar energy spectrum is also depicted in Figure 2. The reflective result shows that the MOS-structure solar cell with a 50-nm ITO/10-nm SiO_2_ layer exhibited good antireflective (AR) performance and that the low reflection band of the reflective spectrum matched the high energy band of the solar energy spectrum. The average weighted reflectance of the MOS-structure solar cell was about 20.27%, which was calculated for wavelengths ranging from 350 to 1,100 nm. The EQE responses of the bare solar cell and the MOS-structure solar cell with a 50-nm ITO/10-nm SiO_2_ AR coating are shown in Figure 3. For the bare solar cell, the peak EQE value of 59% was achieved at approximately 650 nm, but the value declined beyond a wavelength of 900 nm because of reduced absorption at long wavelengths and low minority carrier diffusion lengths. At the wavelength of 1,175 nm, no incident light is absorbed below the band gap of silicon materials, so the EQE is zero at this wavelength. In addition, since the short wavelengths incident lights are absorbed very close to the surface and exhibited a high reflection loss for a bare solar cell, the photo-carriers generated near the surface had high surface recombination losses for a bare solar cell. Thus, the EQE values for wavelengths below 450 nm were lower for a bare solar cell, declining down to 5% at a wavelength of 350 nm. In contrast to the bare solar cell, a peak EQE value of 78% at approximately 650 nm and higher EQE values in the 400 to 1,100 nm range of wavelengths were obtained for the MOS-structure solar cell with a 50-nm ITO/10-nm SiO_2_ AR coating. The EQE response is consistent with the optical reflectance measurement in this study.

**Figure 4:**
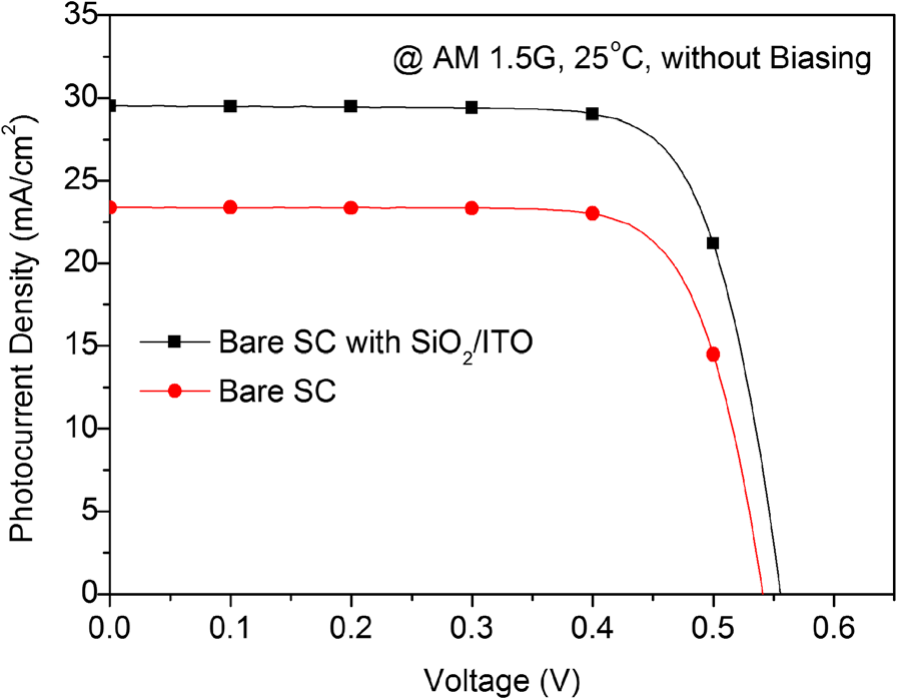
Photovoltaic J-V curves of the bare solar cell and the MOS-structure solar cell without voltage biasing on the ITO electrode (at 0 V).

The photovoltaic J-V curves of the bare solar cell and the MOS-structure solar cell without voltage biasing on the ITO electrode (at 0 V) are shown in Figure 4. Under one-sun AM 1.5G illumination, the bare solar cell had an *J*_*sc*_ of 23.38 mA/cm^2^, an open-circuit voltage (*V*_*oc*_) of 540.8 mV, and a conversion efficiency (*η*) of 9.64%. On the other hand, the *J*_*sc*_ was increased to 29.51 mA/cm^2^, *V*_*oc*_ was increased to 555.6 mV, and *η* was increased to 12.42% after a 50-nm ITO/10-nm SiO_2_ AR coating was deposited on the bare cell. The enhancements of *η* and *J*_*sc*_ of 28.8% and 26.22%, respectively, are attributed to the reduction of the optical reflection loss and surface recombination. The increase in *J*_*sc*_ was consistent with the enhanced EQE because the photocurrent generated was proportional to the EQE value of the photovoltaic devices.

**Figure 5:**
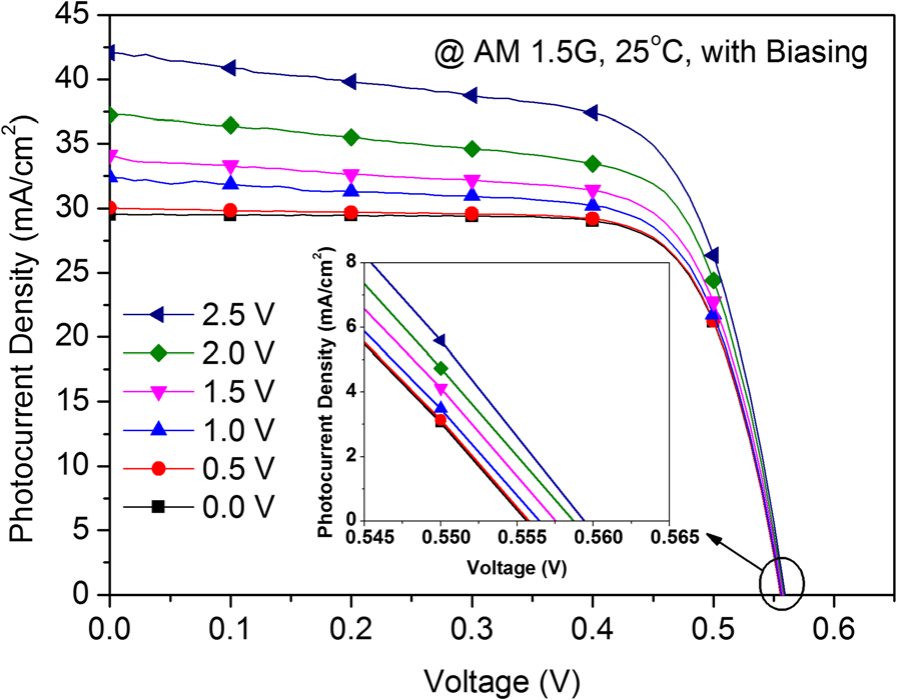
Photovoltaic J-V curves of the MOS-structure solar cell as a function the ITO biasing voltage.

**Figure 6:**
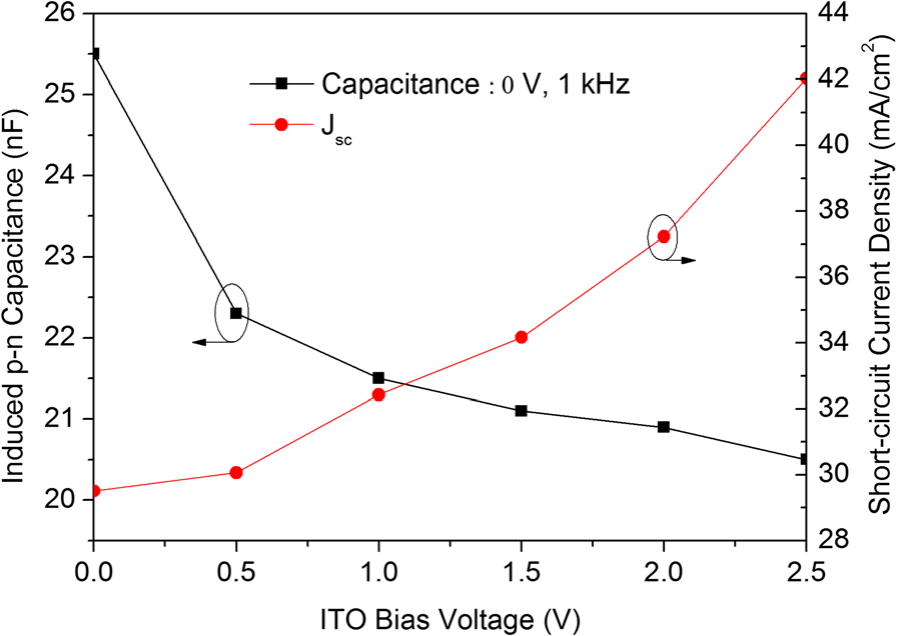
The induced p-n junction capacitance as a function of the ITO biasing voltage.

**Figure 7:**
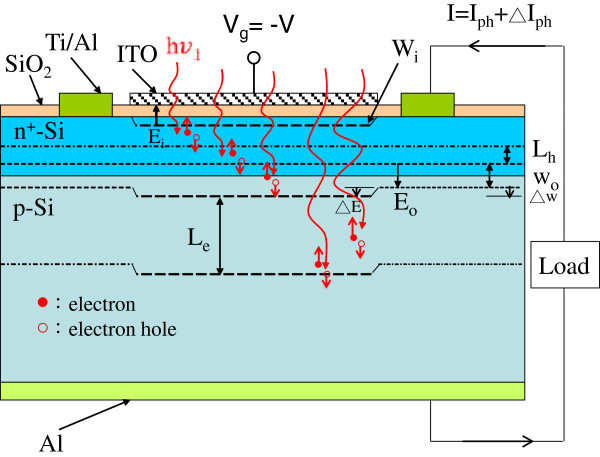
**Schematic of an MOS-structure silicon solar cell for the built-in electric field (E**_**0**_ **+ ∆E) and depletion width (W**_**0**_ **+ ∆W) of the p-n junction under the ITO-electrode dependent on the applied biasing voltage.**

To characterize the degree to which the performance of the MOS-structure solar cell was dependent on the biasing effects, the photovoltaic J-V curves and the induced p-n junction capacitance were measured from 0 to 2.5 V. The photovoltaic J-V curves of the MOS-structure solar cell as a function the ITO biasing voltage are displayed in Figure 5 and summarized in Table 1. The *J*_*sc*_ increased significantly as the biasing voltage was increased; the *V*_*oc*_, in contrast, was much less increased from 555.6 to 559.4 mV as shown in the inset of Figure 5. At 2.5 V, a *J*_*sc*_ of (42.03 mA/cm^2^), a *V*_*oc*_ of 559.4 mV, and a *η* of 15.72% were achieved. This meant that compared to the MOS-structure with 0 V biasing, improvements of *J*_*sc*_ by 42.43% and *η* by 26.57% were obtained due to the biasing effect. The improvement in *η* was less than the improvement in *J*_*sc*_ due to the *FF* being degraded from 0.76 to 0.67. Figure 6 shows the MOS-structure solar cell's induced p-n junction capacitance as a function of the ITO biasing voltage. The p-n junction capacitance at 0 V was about 25.4 nF. However, as the ITO biasing voltage was increased from 0 to 2.5 V, the induced p-n junction capacitance was gradually decreased from 25.4 to 20.5 nF. In general, the junction capacitance (*C*_*j*_) of a p-n device is inversed to the depletion width (*W*) which given by the equation

$$C_{j}=\frac{\epsilon_{s}A}{W}$$

where *ϵ*_*s*_ is permittivity of silicon semiconductor and *A* is the device area. Thus, the decreased in capacitance (as shown in Figure 6) means that the depletion width was increased. Therefore, the absorbed volume would be increased from *V*_*0*_ (= *AW*_*0*_) to *V* (= *V*_*0*_*+ ∆V* = *A* (*W*_*0*_*+ ∆W*)) and the built-in electric field increased from *E*_*0*_ to *E* (= *E*_*0*_*+ ∆E*) because the depletion width of the p-n junction under the ITO-electrode was increased from *W*_*0*_ to *W* (= *W*_*0*_ + *∆W*), as shown in Figure 7, when the biasing voltage increased from 0 to an applied voltage V, where *V*_*0*_, *E*_*0*_, and *W*_*0*_ are the absorbed volume, built-in electric field, and depletion width at zero voltage biasing, respectively, and *∆W* is the induced depletion width. The increased built-in electric field can be obtained from the equation

$$\Delta E=\frac{qN_{a}\Delta W}{A\epsilon_{s}}$$

where *q* is elemental charge and *N*_*a*_ is the dopant concentration of the base layer of proposed solar cell. The induced depletion width can be obtained from the equation

$$\Delta W=\frac{\epsilon_{s}A}{C_{0}-C\left(V\right)}$$

where *C*_*0*_ is the junction capacitance measured at 0 V and C(V) is the capacitance measured at a applied voltage V.

**Table 1:** Photovoltaic performances of the MOS-structure solar cell as a function of the ITO biasing voltage

	**Bare SC**	**MOS SC**					
		**0 V**	**0.5 V**	**1 V**	**1.5 V**	**2 V**	**2.5 V**
*J*_*sc*_ (mA/cm^2^)	23.38	29.51	30.06	32.43	34.17	37.23	42.03
*V*_*oc*_ (mV)	540.8	555.6	555.8	556.5	557.6	558.4	559.4
*FF*	0.76	0.76	0.75	0.71	0.70	0.68	0.67
*η* (%)	9.64	12.42	12.49	12.85	13.34	14.07	15.72

In conclusion, the *J*_*sc*_ and *η* of the proposed MOS-structure solar cell with a biasing voltage were increased because more photo-carriers were generated in a largely absorbed volume and because the transport and collection of the carriers were more effectively due to the electrical biasing effects.

## Conclusions

In this study, high performance enhancement of an ITO/oxide/p-n-semiconductor MOS-structure silicon solar cell with a voltage biasing on the ITO-electrode was experimentally demonstrated. The absorbed volume and built-in electric field in the solar cell were increased due to the biasing effects. Compared to the MOS-structure solar cell without biasing, conversion efficiency enhancement of 26.57% (from 12.42% to 15.72%) and short-circuit current density enhancement of 42.43% (from 29.51 to 42.03 mA/cm^2^) were obtained when the proposed MOS-structure solar cell was biased at 2.5 V.

## Competing interests

The authors declare that they have no competing interests.

## Authors’ contributions

WJH figured out the mechanism about this research, analyzed, and organized the article. MCH, YYL, ZFH, and CJL did the solar cells fabrication, photovoltaic I-V, and C-V and EQE measurements. All authors read and approved the final manuscript.
